# Utility of vaginal vault cytology in the local recurrence of cervical cancer

**DOI:** 10.1186/s12905-023-02371-7

**Published:** 2023-04-20

**Authors:** Kazuto Nakamura, Soichi Yamashita, Keiko Kigure, Toshio Nishimura, Ikuro Ito, Anri Azuma, Kohshiro Nakao, Ken Ando, Tatsuya Kanuma

**Affiliations:** 1 Department of Gynecology, Gunma Prefectural Cancer Center, Ota, Japan; 2 Department of Obstetrics and Gynecology, Takasaki General Medical Center, Takasaki, Japan; 3 Department of Obstetrics and Gynecology, Gunma University, Maebashi, Japan; 4 Department of Radiation Oncology, Gunma University, Maebashi, Japan

**Keywords:** Cervical cancer, Surveillance, Local recurrence, Papanicolaou smear test

## Abstract

**Background:**

In Japan, 8000 women were newly diagnosed with cervical cancer in 2018. The healthcare insurance policy in Japan allows physicians to utilize vaginal volt cytology tests and serum biomarker measurement at every visit and imaging analysis at an adequate interval with screening for recurrence after initial treatment. However, the major surveillance guidelines published in the United States and European countries recommend focusing on pelvic examinations and symptom reviews to avoid unnecessary tests. This study aimed to reassess the benefits of standard surveillance methods adopted in this study by retrospective analysis.

**Methods:**

From January 2009 to December 2015, the medical records of patients with recurrence who were initially diagnosed with International Federation of Gynecology and Obstetrics stage I–III cervical cancer were collected for this study. Clinicopathological data were statistically analyzed to identify significant factors. In the first 2 years, the patients underwent regular surveillance, including pelvic examination, serum tumor marker tests, vaginal vault cytology every 1–3 months, and imaging analysis at 6- to 12-month intervals. In the following 2 years, the patients received a regular check with the same methods every 4 months and an annual imaging analysis. Afterward, the patients had regular screening every 6 to 12 months.

**Results:**

In the study period, 84 of the 981 patients experienced recurrence, and 88.1% had an asymptomatic recurrence. The disease-free interval was not related to the recurrence site. In univariate analysis, primary treatment, recurrence site, and diagnostic method were significant factors for survival outcomes. In contrast, multivariate analysis indicated that only primary treatment was a significant factor. In patients with local recurrence, multivariate analysis demonstrated that radiation as salvage therapy was an independent predictive factor for overall survival after recurrence.

**Conclusions:**

In this retrospective study, routine imaging analysis and serum biomarker measurement did not contribute to patient prognosis after recurrence. In contrast, vaginal vault cytology can improve survival after recurrence in some patients. Tailored surveillance methods based on individual disease conditions and treatment modalities can improve post-recurrent survival outcomes.

**Supplementary Information:**

The online version contains supplementary material available at 10.1186/s12905-023-02371-7.

## Introduction

The number of patients with cervical cancer has been gradually increasing in Japan since 2000. Approximately 8000 patients were newly diagnosed with cervical cancer in 2018 [1]. In addition, the age-adjusted peak prevalence of cervical cancer has shifted to a younger age of approximately 40 years over the last decade. Because approximately 75% of the patients are categorized with stage I and II cervical cancer, most patients are expected to have a favorable prognosis. However, some women, especially those in the advanced stage, remain at risk of tumor recurrence. Post-primary treatment surveillance is assumed to detect early recurrence, which can result in prolonged survival and improved quality of life by utilizing adequate salvage therapy and supportive care. However, different institutions and countries have variable protocols for surveillance because of a lack of solid evidence on surveillance methods and follow-up intervals.

In contrast to pelvic sidewall and distant metastasis, local recurrence is amenable to curative therapy [2, 3]. Cervicovaginal cytology has been used to detect local recurrence [4], but its detection rate is low [5, 6]. Thus, the Society of Gynecologic Oncology (SGO) has recommended cytological evaluations should be limited to once per year [7]. Patients may also desire to undergo imaging analyses, including computed tomography (CT), magnetic resonance imaging (MRI), and positron-emission tomography (PET) scan to test for asymptomatic recurrence. However, in terms of cost–benefit balance, no study has reported that regular surveillance with imaging analysis has not improved survival after recurrence because only local recurrence is potentially curable. Thus, the SGO and European Society for Medical Oncology (ESMO) guidelines do not recommend routine surveillance with imaging analysis, except for limited cases [7, 8]. Serum squamous cell carcinoma (SCC) for squamous cell tumors and cancer antigen 125 (CA125) levels for adenocarcinoma in cervical cancer are also used to evaluate disease stage, response to therapy, and relapse of tumors. Increasing serum SCC and CA125 levels can suggest the recurrence of squamous cell tumors and adenocarcinoma prior to clinical symptoms [9, 10]. However, asymptomatic recurrence diagnosed by elevated serum SCC and CA125 levels does not contribute to improved survival outcomes [11].

In Japan, nearly all patients are covered by public health insurance, which applies to the majority of medical charges, allowing physicians to perform intensive surveillance using pelvic examination, the Papanicolaou (Pap) smear test, measurement of serum tumor antigen levels at every visit, and periodic imaging analysis, including CT, MRI, and PET scan, for patients with cervical cancer, even without any symptoms.

As mentioned above, the guidelines released from the United States and European countries recommend concentrating on signs and symptoms to reduce unnecessary examinations and save costs. However, controversy remains regarding routine surveillance using vaginal vault cytology and imaging analysis. In this study, we first evaluated the intensive surveillance protocol adopted in Japan by retrospectively analyzing data, including surveillance methods to detect recurrence, recurrence sites, and survival periods after recurrence. We also aimed to examine factors that positively improve survival outcomes after recurrence. Ultimately, we intend to reconsider the surveillance method for routine follow-up based on the results of this study.

## Methods

### Ethical consideration

This study was approved by the Ethics Committee of Gunma Prefectural Cancer Center (approval # 405–31,012). The study protocol was approved by the Gunma University Hospital Clinical Research Review Board and Ethics Review Committee of the National Hospital Organization Takasaki General Medical Center. All methods were performed in accordance with relevant guidelines and regulations (Declaration of Helsinki).

According to the ethical guidelines for medical and health research involving human subjects in Japan, informed consent is not required for medical studies that use only medical records without human samples, and the analysis is conducted with anonymized data. Thus, informed consent was waived by The Ethics Committees of Gunma Prefectural Cancer. However, all patients were provided the right to withdraw their consent for the use of data using the opt-out method on the Gunma Prefectural Cancer Center website in 2022. The Ethics Committees of Gunma Prefectural Cancer Center approved the opt-out method for obtaining participant consent for this study.

### Study design

The basic study design has been published in our previous surveillance study for endometrial cancer [12]. The medical records were obtained for patients with cervical cancer initially diagnosed according to the International Federation of Gynecology and Obstetrics (FIGO 2018) stage 1-3c2 between 2009 and 2015.

In the first 2 years, the patients underwent regular surveillance, including a pelvic examination, Pap smear test, and serum biomarker test (SCC or CA125) every 1–3 months and imaging analysis at 6- to 12-month intervals. In the following 2 years, regular surveillance was conducted every 4 months with annual imaging analysis. Afterward, the patients received standard surveillance every 6 to 12 months. The medical records of patients who developed recurrent tumors were collected from three institutions and analyzed for this retrospective study. All patients with recurrence in FIGO stage I-3c2 were included.

### Statistical analyses

Demographic data, FIGO stage, histology, primary therapy, method of diagnosis for recurrent tumors, salvage therapy after recurrence, and survival period after recurrence were incorporated into statistical analysis since these factors are potentially involved in evaluating the significance of surveillance methods and survival outcomes. Intervals between visits before the diagnosis of recurrence were stratified into 1, 2, and > 3 months to verify lead-time bias after recurrence. Univariate and multivariate analyses were performed for all patients after recurrence (Table 2). Univariate and multivariate analyses were also conducted for patients with local recurrence (Table 3) since those patients had better overall survival after recurrence (Fig. 2A). The time from recurrence to death was analyzed using a Cox proportional hazards model to calculate hazard ratios (HRs) and 95% confidence intervals (95% CIs) after the proportional hazard test was performed. The included factors were FIGO stage, histology, primary treatment, recurrence site, and diagnosis method at recurrence in Table 1; diagnosis method, initial treatment, and salvage therapy after recurrence in Table 2; and diagnosis method at recurrence, initial treatment, and salvage therapy after recurrence in Table 3. After multicollinearity was evaluated using a variance inflation factor, Cox regression analysis was also performed in multivariate analysis, using a stepwise variable selection method for the factors that showed statistically significant differences in the univariate analysis (Tables 2 and 3). The disease-free interval after primary treatment and the survival curves for overall post-recurrence survival by recurrence site, diagnostic method, or local recurrence were calculated using Kaplan–Meier method and log-rank test. Statistical significance was set at *p* < 0.05; all tests were two-tailed. All statistical analysis were performed using EZR version 1.55 [13].

**Table 1:** Patients characteristics at initial treatment and recurrence

**Characteristics**	**No**	**%**
**Initial cancer**		
**Age**	**26–83 (52)**^**a**^	
**FIGO stage**		
**1a**	**3**	**(3.6)**
** 1b1**	**12**	**(14.3)**
** 1b2**	**4**	**(4.7)**
** 1b3**	**4**	**(4.7)**
** 2a1**	**5**	**(6.0)**
** 2a2**	**2**	**(2.4)**
** 2b**	**10**	**(11.9)**
** 3a**	**2**	**(2.4)**
** 3b**	**3**	**(3.6)**
** 3c1**	**34**	**(40.4)**
** 3c2**	**5**	**(6.0)**
**Histology**		
**squamous**	**49**	**(58.3)**
** adeno**	**24**	**(28.6)**
** adenosquamous**	**5**	**(6.0)**
**Others**	**6**	**(7.1)**
**Primary treatment**		
**Ope**	**15**	**(17.9)**
** Ope + RT**	**6**	**(7.1)**
** Ope + CCRT**	**16**	**(19.0)**
** Ope + Chemo**	**13**	**(15.5)**
** RT**	**9**	**(10.7)**
** CCRT**	**25**	**(29.8)**
**At recurrence**		
**Follow up interval**		
**1 months**	**46**	**(54.7)**
** 2 months**	**24**	**(28.6)**
** 3 > months**	**14**	**(16.7)**
**Recurrence site**		
**Local**	**19**	**(22.6)**
** Pelvic LN**	**9**	**(10.7)**
** Distal LN**	**14**	**(16.7)**
** Distant metastasis**	**27**	**(32.1)**
** Multipe metasitasis**	**15**	**(17.9)**
**Method of diagnosis**		
** Symptom**	**10**	**(11.9)**
** Pap smear**	**13**	**(15.5)**
** Tumor marker**	**19**	**(22.6)**
** Imaging analysis**	**42**	**(50.0)**

^a^Range (median)

*Ope* Operation, *RT* Radiation therapy, *CCRT* Concurrent chemo-radiation therapy, *Chemo* Chemotherapy, *LN* Lymph node

**Table 2:** Univariate and multivariate analyses for survival outcomes after recurrence

	**Univariate**		**Multivariate**	
	**Hazard ratio [95% CI]**	*p*-value^*^	**Hazard ratio [95% CI]**	*p*-value^*^
**FIGO stage**				
**I**	**ref**			
**II**	**1.63 [0.76–3.48]**	**0.211**		
**IIIa and IIIb**	**1.44 [0.41–5.08]**	**0.571**		
**IIIc1 and IIIc2**	**1.76 [0.94–3.31]**	**0.080**		
**Histology**		**0.270**		
**Squamous**	**ref**			
**adeno**	**0.84 [0.47–1.48]**	**0.540**		
**Other**	**1.59 [0.78–3.21]**	**0.200**		
**Primary treatment**				
**Ope**	**ref**		**ref**	
**Ope + Chemo**	**4.09 [1.46–11.4]**	**0.007**	**3.14 [1.05–9.42]**	**0.041**
**Ope + RT or CCRT**	**4.83 [1.86–12.55]**	**0.001**	**4.81 [1.60–14.45]**	**0.005**
**RT or CCRT**	**4.23 [1.70–10.73]**	**0.002**	**4.62 [1.57–13.60]**	**0.005**
**Recurrence site**				
**Local**	**ref**		**Included but not significant in final multivariate analysis**	
**Pelvic LN**	**2.83 [1.07–7.52]**	**0.036**		
**Distal LN**	**1.51 [0.63–3.60]**	**0.353**		
**Distant metastasis**	**2.68 [1.24–5.81]**	**0.012**		
**Multiple metastasis**	**4.70 [1.99–11.07]**	** < 0.001**		
**Diagnosis method at recurrence**				
**Pap Smear**	**ref**		**Included but not significant in final multivariate analysis**	
**Symptom**	**12.43 [3.73–41.46]**	** < 0.001**		
**Tumor Marker**	**4.13 [1.47–11.58]**	**0.007**		
**Imaging analysis**	**2.94 [1.14–7.55]**	**0.025**		

^*^Cox regression analysis was used

*Ope* Operation, *RT* Radiation therapy, *CCRT* Concurrent chemoradiation therapy, *Chemo* Chemotherapy, *LN* Lymph node

**Table 3:** Univariate and multivariate analyses for survival outcomes after recurrence in patients with local recurrence

	**Univariate**		**Multivariate**	
	**Hazard ratio [95% CI]**	*p*-value^*^	**Hazard ratio [95% CI]**	*p*-value^*^
**Diagnosis method at recurrence**				
**Pap Smear**	**ref**		**Included but not significant in final multivariate analysis**	
**Symptom**	**19.10 [1.99–183.40]**	**0.011**		
**Tumor Marker**	**12.03 [0.91–159.10]**	**0.059**		
**Imaging analysis**	**5.69 [1.09–29.77]**	**0.039**		
**Initial treatment**				
**Ope, ope + chemo**	**ref**		**Included but not significant in final multivariate analysis**	
**Ope + RT or CCRT, RT, CCRT**	**5.84 [1.16–29.38]**	**0.030**		
**Salvage therapy after recurrence**				
**RT**	**ref**		**ref**	
**Chemotherapy**	**18.52 [2.15–160]**	**0.008**	**18.52 [2.15–160]**	**0.008**

^*^Cox regression analysis was used

*Ope* Operation, *RT* Radiation therapy, *CCRT* Concurrent chemoradiation therapy, *Chemo* Chemotherapy, *LN* Lymph node

## Results

From January 2009 to December 2015, 981 patients were treated for cervical cancer at three institutions participating in this study. The medical records of 84 patients with recurrent tumors were collected for this study. Recurrence developed for 56% and 79% of the patients within 1 and 2 years, respectively. Overall, 8.6% of the patients suffered from recurrence. The risks of recurrence for each stage were 1.1%, 7.0%, 9.4%, and 17.5% in stages Ia, Ib, II, and III, respectively. The basic characteristics of the patients with recurrent disease are shown in Table 1.

At the initial diagnosis, 44 (52.4%) patients were diagnosed with stage III cervical cancer, and 49 (58.3%) had squamous cell tumors. In the primary treatment, 35 of the 50 patients who underwent surgery as an initial treatment received adjuvant therapy with radiation, concurrent chemoradiation, or chemotherapy. Forty-six (54.7%) patients were diagnosed with recurrence at 1-month follow-up intervals. Nineteen (22.6%) patients had local recurrence; only 10 (11.9%) patients had a symptomatic recurrence.

**Fig. 1:**
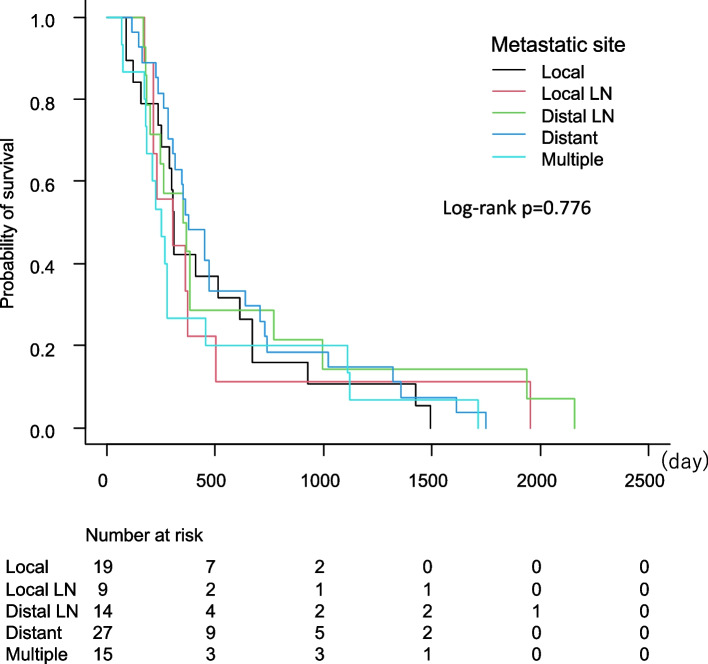
Disease-free interval after primary treatment

**Fig. 2:**
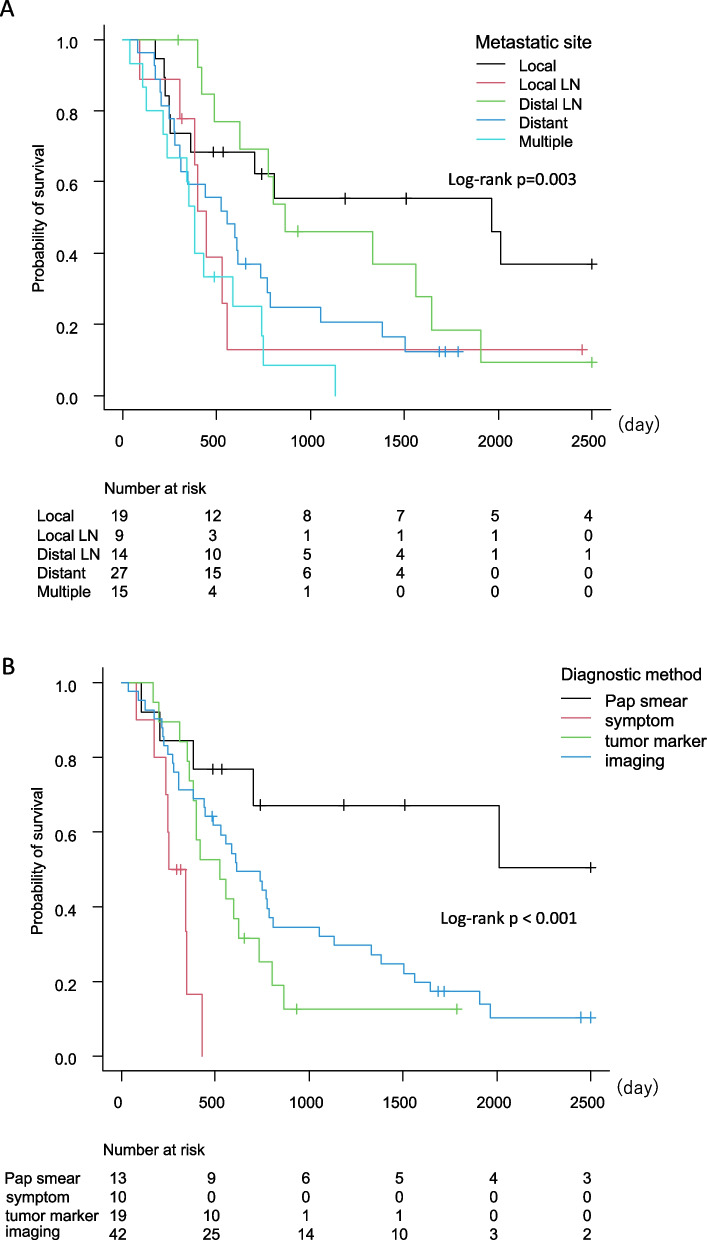
Overall survival after recurrence. **A** Metastatic site, (**B**) diagnostic method

The Kaplan–Meier curve showed the disease-free interval after primary treatment (Fig. 1). No significant difference was found in the recurrence site (*p* = 0776). Patients with local recurrence (Fig. 2A) (*p* = 0.003) or recurrence diagnosis by Pap smear test (Fig. 2B) (*p* < 0.001) had better overall survival after recurrence than those with other sites or diagnosed by other methods. The HRs and 95% CIs for survival after recurrence were calculated using Cox regression analysis (Table 2), which found no significant differences in the FIGO stage and histology. However, primary treatment, recurrence site, and diagnostic method were significant factors. For primary treatment, patients who underwent surgery showed a significantly better prognosis than those who underwent other treatment methods. Patients with local recurrence had better survival outcomes than those with pelvic LN metastasis, distant metastasis, or multiple metastases. Symptoms along with tumor markers and imaging analysis for the diagnostic method at recurrence had poor survival against Pap smear. Multivariate analysis found that primary treatment was the only prognostic factor.

**Fig. 3:**
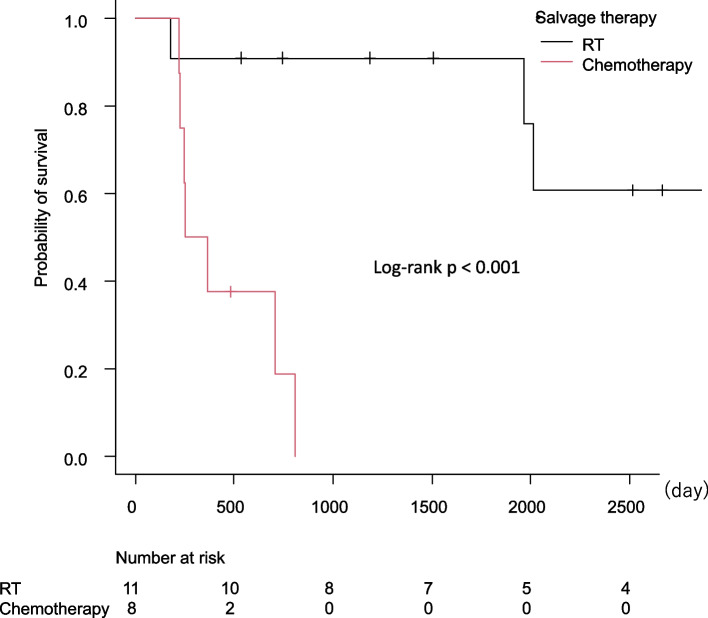
Overall survival after local recurrence by salvage therapy

As patients with local recurrence had better outcomes after recurrence, survival probabilities were compared using the Kaplan–Meier curve (Fig. 3), demonstrating that patients who were treated with radiotherapy had a significantly better prognosis than those who were treated with chemotherapy (*p* < 0.001). We further investigated factors affecting survival after recurrence (Table 3). In the diagnostic method at recurrence, the Pap smear showed better survival outcomes against symptoms and imaging analysis. Patients without radiation at initial treatment and with radiation treatment for salvage therapy had better prognoses compared with the others in the univariate analysis; radiation therapy after recurrence was the only prognostic factor in the multivariate analysis.

## Discussion

In this retrospective multicenter study, we have presented that patients with local recurrence who were not treated with radiation therapy in the primary treatment had better overall survival. For those patients, the Pap smear test was an effective method for detecting recurrent tumors. Moreover, patients with local recurrence who could receive radiation therapy as salvage therapy had a chance of a complete cure.

Most patients with recurrent cervical cancer have a poor prognosis, although some specific patients demonstrate long-term survival after recurrence. Patients expect posttreatment surveillance to detect recurrence at a significantly early stage that is amenable to curative treatment. However, surveillance methods and concepts vary among countries and institutions and even among physicians in the same hospital, owing to a lack of definitive evidence. This study analyzed the most intensive surveillance methods adopted in daily clinical practice under Japan’s public health insurance coverage.

The concept of intense surveillance leads to survival benefits and a better quality of life even after recurrence. In this study, nearly all the patients were observed by gynecological oncologists during the surveillance period. According to the intensive surveillance protocol analyzed in this study, recurrences at 1 and 2 years were diagnosed at 56 and 79%, respectively, which is consistent with previous reports [5, 14, 15]; no difference was found in the disease-free interval by the metastatic site (Fig. 1). In fact, 54.7% of recurrences were diagnosed within the 1-month follow-up interval. Moreover, only 16.7% of patients had more than a 3-month follow-up interval, and 90.5% had asymptomatic recurrence (Table 1). To the best of our knowledge, this rate is the highest for the detection of asymptomatic recurrence, overall survival after recurrence, asymptomatic recurrence, and symptomatic recurrence (HR, 4.28; 95% CI, 1.87–9.78; *p* < 0.001). A recent study with a large cohort of 4343 patients with stage I and II cervical cancer demonstrated that asymptomatic recurrence was a significant factor for survival after recurrence [16]. In contrast, a review of 17 retrospective studies in which follow-up visit intervals were every 3–4 months in the first 2 years did not show any benefit in detecting asymptomatic recurrence [17]. A better prognosis can be achieved by lead-time bias. However, our study can exclude lead-time bias regarding better survival after recurrence in asymptomatic recurrence because most patients were diagnosed at the 1–2-month visit interval.

Imaging analyses, including CT, MRI, and PET, were utilized to detect asymptomatic recurrence for routine surveillance. In this study, 42 (50.0%) patients were diagnosed with tumor recurrence using imaging analyses (Table 1). Among these patients, 27 had distant metastasis, and three with solitary lung metastasis were completely cured by surgery. Other studies have also reported successful treatment of isolated pulmonary recurrence [6, 18]. An Italian multicenter retrospective study demonstrated that 80–90% of distant and lymph node metastases were diagnosed by imaging analysis [19], which means that imaging analysis is more sensitive for identifying distant metastasis. Although a few patients, including our cases, benefit from routine imaging analyses to detect asymptomatic recurrence in distant regions, the SGO does not recommend routine imaging because of both the cost–benefit aspect and low yield in the diagnosis of asymptomatic recurrence [20]. In addition, an annual CT scan might increase radiation-related cancer risk [21]. Thus, radiological modalities should be avoided without a clear indication.

Serum biomarkers, such as SCC and CA-125, have been used to assess tumor response to treatment [22] and detect recurrence by routine surveillance [23]. An earlier report showed that serum SCC had 79% sensitivity for detecting recurrence [24]. In this study, 22.6% of patients were diagnosed with recurrent tumors by elevated tumor marker levels (Table 1), but this did not contribute to improved survival after recurrence (Fig. 2B). Consistent with our study, a Dutch study demonstrated that elevated serum SCC levels preceded clinical symptoms, resulting in no correlation with survival outcomes [9]. Collectively, owing to the lack of supportive evidence for measuring serum biomarkers, routine surveillance may omit serum biomarker measurement unless it is useful for judging the response to treatment against tumors by the primary treatment.

Consistent with previous publications, this study showed that local recurrence had a better prognosis than local lymph nodes, distal lymph nodes, or distant metastases (Fig. 2A). Even in this study, seven patients with multiple metastases had local recurrence. Although none of our patients diagnosed with only local recurrence had pelvic exenteration for salvage therapy, the literature describes that one-third of cases were found to be contraindicated for pelvic exenteration at laparotomy due to unpredictable spread of tumor-like dissemination [25]. Thus, careful evaluation of the tumor condition should be considered before deciding on treatment modality since aggressive surgery can attenuate a patient’s quality of life.

Pelvic examination and vaginal vault cytology have been performed for routine surveillance. A systemic review found that the detection rate of asymptomatic recurrence by Pap smear was remarkably low (6% of the median detection rate) [17], and physical examinations found local recurrence at a higher rate than cytological evaluation [6]. Moreover, low-grade results in a Pap smear followed by colposcopy demonstrated less effectiveness for recurrence detection [20]. However, in our study, univariate analysis showed that the Pap smear in the diagnostic method was a significant factor for survival after recurrence (Table 2 and Fig. 2B). One could think that the vaginal suturing method of hysterectomy can affect the diagnosis of recurrence at the vaginal cuff. An introflexion suture could facilitate a Pap smear to detect the recurrent tumor. However, the suturing method had not helped the detection of local recurrence since all cases of hysterectomy in our study were confirmed to have received extroflection sutures. Radiation therapy can produce fibrotic changes in the vaginal mucosa, possibly resulting in a higher rate of abnormal Pap smear results [26]. In the current study, seven of the nine patients with local recurrence diagnosed by Pap smear did not receive radiation therapy in the initial treatment, possibly explaining why the Pap smear contributed to accurate recurrence diagnoses.

Similar to other reports [27, 28], radiation history as initial treatment in univariate analysis and radiation therapy as salvage therapy in multivariate analysis are significant factors for survival after local recurrence (Table 3). The options for salvage therapy vary among patients, depending on the histology of the tumor, site of recurrence, and patient performance status. In a different set of patients with isolated pelvic tumors previously treated with radiation therapy, no statistical difference was found in cumulative distant metastasis and overall survival between chemotherapy and surgery, including radical hysterectomy and pelvic exenteration for salvage therapy [29]. In contrast, pelvic exenteration can have a prognostic effect on patients contraindicated for radiation therapy, with 5-year survival rates between 30 and 40% [30, 31]. As observed in the current study, patients who received chemotherapy for salvage therapy had poor outcomes (Fig. 3). Thus, aggressive surgery is considered for patients with intolerable risks associated with re-radiation therapy. However, the difficulty in presurgically assessing resectability and the relatively high perioperative morbidity and mortality discourage the indication of radical surgery.

The prognostic factors of local recurrence associated with survival after salvage radiation include disease-free interval, histology, site of recurrence (i.e., around the vaginal apex or pelvic sidewall), and tumor size. Among these factors, tumor size and location highly predict curative treatment [32, 33]. In this study, the outcomes of patients who received radiation therapy for local recurrence were substantially good, as confirmed by the fact that the median overall survival was not reached at the time of data fixation (Fig. 3). The main purpose of intensive surveillance is to detect tumors at an early stage of recurrence when they are amenable to curative therapy. In this regard, the Pap smear, which effectively identifies asymptomatic recurrence in patients without a history of radiation, may play the most important role among intensive surveillance methods, which can eventually benefit patients by allowing the prescription of curable radiation therapy instead of aggressive surgery. Moreover, early detection of asymptomatic recurrence through intensive surveillance may provide survivors with psychological support and effective symptom control.

This study was conducted using multicenter data to lower patient selection bias. However, this study has some limitations, including a small sample size and that the retrospective nature of the study may generate bias. Although this study presents statistical significance with the sample size, larger study cohorts can provide more precise data and smaller confidence intervals. Clinical outcomes after aggressive surgery were not evaluated because none of the patients underwent this surgery in this study period. The results of our study require further validation in future studies with larger cohorts and randomized controlled trials.

## Conclusions

The results of this study confirm that routine imaging analysis and biomarker measurement do not contribute to a patient’s prognosis after recurrence. However, the Pap smear test can improve survival after recurrence in some patient groups. Further developments in diagnostic modalities will help tailor surveillance methods for individual patients.

## Supplementary Information

**Additional file 1.**

## Data Availability

The data used in this study are available from the corresponding author upon reasonable request.

## Abbreviations

Pap smear test: Papanicolaou smear test
SGO: Society of Gynecologic Oncology
CT: Computed tomography
MRI: Magnetic resonance imaging
PET: Positron emission tomography
ESMO: European Society for Medical Oncology
FIGO: International Federation of Gynecology and Obstetrics

## References

[1] Yoshino K, Kurita T, Takahashi F, Nagase S (2022). Board members of the Committee on Gynecologic Oncology of the Japan Society of O, Gynecology: Annual report of the committee on gynecologic oncology, the Japan Society of Obstetrics and Gynecology: Annual patient report for 2019 and annual treatment report for 2014. J Obstet Gynaecol Res.

[2] Krebs HB, Helmkamp BF, Sevin BU, Poliakoff SR, Nadji M, Averette HE (1982). Recurrent cancer of the cervix following radical hysterectomy and pelvic node dissection. Obstet Gynecol.

[3] Potter ME, Alvarez RD, Gay FL, Shingleton HM, Soong SJ, Hatch KD (1990). Optimal therapy for pelvic recurrence after radical hysterectomy for early-stage cervical cancer. Gynecol Oncol.

[4] Koh WJ, Greer  BE, Abu-Rustum NR, Apte SM, Campos SM, Cho KR, Chu C, Cohn D, Crispens MA, Dorigo O (2015). Cervical Cancer, Version 2.2015. J Natl Compr Canc Netw.

[5] Bodurka-Bevers D, Morris M, Eifel PJ, Levenback C, Bevers MW, Lucas KR, Wharton JT (2000). Posttherapy surveillance of women with cervical cancer: an outcomes analysis. Gynecol Oncol.

[6] Elit L, Fyles AW, Oliver TK, Devries-Aboud MC, Fung-Kee-Fung M (2010). members of the Gynecology Cancer Disease Site Group of Cancer Care Ontario’s Program in Evidence-Based C: Follow-up for women after treatment for cervical cancer. Curr Oncol.

[7] Salani R, Khanna N, Frimer M, Bristow RE, Chen LM (2017). An update on posttreatment surveillance and diagnosis of recurrence in women with gynecologic malignancies: Society of Gynecologic Oncology (SGO) recommendations. Gynecol Oncol.

[8] Marth C, Landoni F, Mahner S, McCormack M, Gonzalez-Martin A, Colombo N, Committee EG (2017). Cervical cancer: ESMO Clinical Practice Guidelines for diagnosis, treatment and follow-up. Ann Oncol.

[9] Esajas MD, Duk JM, de Bruijn HW, Aalders JG, Willemse PH, Sluiter W, Pras B, ten Hoor K, Hollema H, van der Zee AG (2001). Clinical value of routine serum squamous cell carcinoma antigen in follow-up of patients with early-stage cervical cancer. J Clin Oncol.

[10] Tabata T, Takeshima N, Tanaka N, Hirai Y, Hasumi K (2000). Clinical value of tumor markers for early detection of recurrence in patients with cervical adenocarcinoma and adenosquamous carcinoma. Tumour Biol.

[11] Salvatici M, Achilarre MT, Sandri MT, Boveri S, Vanna Z, Landoni F (2016). Squamous cell carcinoma antigen (SCC-Ag) during follow-up of cervical cancer patients: Role in the early diagnosis of recurrence. Gynecol Oncol.

[12] Nakamura K, Kitahara Y, Yamashita S, Kigure K, Ito I, Nishimura T, Azuma A, Kanuma T (2022). Reassessment of intensive surveillance practices adopted for endometrial cancer survivors. BMC Womens Health.

[13] Kanda Y (2013). Investigation of the freely available easy-to-use software ‘EZR’ for medical statistics. Bone Marrow Transplant.

[14] Hillesheim I, Limone GA, Klimann L, Monego H, Appel M, de Souza A, Dos Reis R (2017). Cervical Cancer Posttreatment Follow-up: Critical Analysis. Int J Gynecol Cancer.

[15] Taarnhoj GA, Christensen IJ, Lajer H, Fuglsang K, Jeppesen MM, Kahr HS, Hogdall C (2018). Risk of recurrence, prognosis, and follow-up for Danish women with cervical cancer in 2005–2013: A national cohort study. Cancer.

[16] Cibula D, Dostalek L, Jarkovsky J, Mom CH, Lopez A, Falconer H, Scambia G, Ayhan A, Kim SH, Isla Ortiz D (2022). Post-recurrence survival in patients with cervical cancer. Gynecol Oncol.

[17] Elit L, Fyles AW, Devries MC, Oliver TK, Fung-Kee-Fung M (2009). Gynecology Cancer Disease Site G: Follow-up for women after treatment for cervical cancer: a systematic review. Gynecol Oncol.

[18] Samlal RA, Van Der Velden J, Van Eerden T, Schilthuis MS, Gonzalez Gonzalez D, Lammes FB (1998). Recurrent cervical carcinoma after radical hysterectomy: an analysis of clinical aspects and prognosis. Int J Gynecol Cancer.

[19] Zola P, Fuso L, Mazzola S, Piovano E, Perotto S, Gadducci A, Galletto L, Landoni F, Maggino T, Raspagliesi F (2007). Could follow-up different modalities play a role in asymptomatic cervical cancer relapses diagnosis? An Italian multicenter retrospective analysis. Gynecol Oncol.

[20] Rimel  BJ, Burke WM, Higgins RV, Lee PS, Lutman CV, Parker  L (2015). Improving quality and decreasing cost in gynecologic oncology care. Society of gynecologic oncology recommendations for clinical practice. Gynecol Oncol.

[21] Wen JC, Sai V, Straatsma BR, McCannel TA (2013). Radiation-related cancer risk associated with surveillance imaging for metastasis from choroidal melanoma. JAMA Ophthalmol.

[22] Yoon SM, Shin KH, Kim JY, Seo SS, Park SY, Kang S, Cho KH (2007). The clinical values of squamous cell carcinoma antigen and carcinoembryonic antigen in patients with cervical cancer treated with concurrent chemoradiotherapy. Int J Gynecol Cancer.

[23] Jeong BK, Huh SJ, Choi DH, Park W, Bae DS, Kim BG (2013). Prognostic value of different patterns of squamous cell carcinoma antigen level for the recurrent cervical cancer. Cancer Res Treat.

[24] Forni F, Ferrandina G, Deodato F, Macchia G, Morganti AG, Smaniotto D, Luzi S, D’Agostino G, Valentini V, Cellini N (2007). Squamous cell carcinoma antigen in follow-up of cervical cancer treated with radiotherapy: evaluation of cost-effectiveness. Int J Radiat Oncol Biol Phys.

[25] Estape R, Angioli R (1999). Surgical management of advanced and recurrent cervical cancer. Semin Surg Oncol.

[26] Rimel BJ, Ferda A, Erwin J, Dewdney SB, Seamon L, Gao F, DeSimone C, Cotney KK, Huh W, Massad LS (2011). Cervicovaginal cytology in the detection of recurrence after cervical cancer treatment. Obstet Gynecol.

[27] Qiu JT, Abdullah NA, Chou HH, Lin CT, Jung SM, Wang CC, Chen MY, Huang KG, Chang TC, Lai CH (2012). Outcomes and prognosis of patients with recurrent cervical cancer after radical hysterectomy. Gynecol Oncol.

[28] Yoshida K, Kajiyama H, Utsumi F, Niimi K, Sakata J, Suzuki S, Shibata K, Kikkawa F (2018). A post-recurrence survival-predicting indicator for cervical cancer from the analysis of 165 patients who developed recurrence. Mol Clin Oncol.

[29] Lin AJ, Ma S, Markovina S, Schwarz J, Mutch DG, Powell MA, Grigsby PW (2019). Clinical outcomes after isolated pelvic failure in cervical cancer patients treated with definitive radiation. Gynecol Oncol.

[30] Schmidt AM, Imesch P, Fink D, Egger H (2012). Indications and long-term clinical outcomes in 282 patients with pelvic exenteration for advanced or recurrent cervical cancer. Gynecol Oncol.

[31] Chiantera V, Rossi M, De Iaco P, Koehler C, Marnitz S, Ferrandina G, Legge F, Parazzini F, Scambia G, Schneider A (2014). Survival after curative pelvic exenteration for primary or recurrent cervical cancer: a retrospective multicentric study of 167 patients. Int J Gynecol Cancer.

[32] Ito H, Shigematsu N, Kawada T, Kubo A, Isobe K, Hara R, Yasuda S, Aruga T, Ogata H (1997). Radiotherapy for centrally recurrent cervical cancer of the vaginal stump following hysterectomy. Gynecol Oncol.

[33] Ijaz T, Eifel PJ, Burke T, Oswald MJ (1998). Radiation therapy of pelvic recurrence after radical hysterectomy for cervical carcinoma. Gynecol Oncol.

